# Childhood and adult tuberculosis in a rural hospital in Southeast Ethiopia: a ten-year retrospective study

**DOI:** 10.1186/1471-2458-10-215

**Published:** 2010-04-27

**Authors:** Jose Manuel Ramos, Francisco Reyes, Abraham Tesfamariam

**Affiliations:** 1 Gambo General Rural Hospital, POB 121, Shashemane, Ethiopia; 2 Infectious Diseases Unit, Hospital General Universitario de Elche, Camí L'Almazara, 11, 03203 Elche, Alicante, Spain

## Abstract

**Background:**

Many DOTS experiences in developing countries have been reported. However, experience in a rural hospital and information on the differences between children and adults are limited. We described the epidemiology and treatment outcome of adult and childhood tuberculosis (TB) cases, and identified risk factors associated with defaulting and dying during TB treatment in a rural hospital over a 10-year period (1998 to 2007).

**Methods:**

Retrospective data collection using TB registers and treatment cards in a rural private mission hospital. Information was collected on number of cases, type of TB and treatment outcomes using standardised definitions.

**Results:**

2225 patients were registered, 46.3% of whom were children. A total of 646 patients had smear-positive pulmonary TB (PTB), [132 (20.4%) children]; 816 had smear-negative PTB [556 (68.2%) children], and 763 extra-PTB (EPTB) [341 (44.8%) children]. The percentage of treatment defaulters was higher in paediatric (13.9%) than in adult patients (9.3%) (p = 0.001). The default rate declined from 16.8% to 3.5%, and was independently positively associated with TB meningitis (AOR: 2.8; 95% CI: 1.2-6.6) and negatively associated with smear-positive PTB (AOR: 0.6; 95% CI: 0.4-0.8). The mortality rate was 5.3% and the greatest mortality was associated with adult TB (AOR: 1.7; 95% CI: 1.1-2.5), TB meningitis (AOR: 3.6; 95% CI:1.2-10.9), and HIV infection (AOR: 4.3; 95% CI: 1.9-9.4). Decreased mortality was associated with TB lymphadenitis (AOR: 0.24; 95% CI: 0.11-0.57).

**Conclusion:**

(1) The registration of TB cases can be useful to understand the epidemiology of TB in local health facilities. (2) The defaulter and mortality rate of childhood TB is different to that of adult TB. (3) The rate of defaulting from treatment has declined over time.

## Background

Tuberculosis (TB) is one of the leading causes of morbidity and death in adults in sub-Saharan African countries. Ethiopia is among the 22 countries with the highest TB burden in the world. Detection and treatment of new cases in Directly Observed Treatment Short course chemotherapy (DOTS) programmes is believed to be the most valuable strategy for TB control [1]. By the end of 2006, 184 countries had adopted and implemented DOTS programmes. In 1992 the National Tuberculosis and Leprosy Control Programme (TLCP) and DOTS strategy were established in Ethiopia, with guidelines that make it necessary to fill out a TB register [2,3]. A DOTS strategy was introduced in the tuberculosis control programme at Gambo Rural Hospital (GRH) in 1996. In Ethiopia, the resources for accurate TB diagnosis are basic, and culture and drug susceptibility testing for *Mycobacterium tuberculosis *are not performed routinely.

Children account for a substantial proportion of the global burden of tuberculosis [4]. Childhood TB nevertheless remains neglected for various reasons, mainly the difficulty in diagnosing pulmonary TB, the lack of scientific studies on childhood TB, the largely unknown outcome of children with TB, and the dogma that childhood TB is not important in TB control [4-6]. Moreover, children in TB-endemic areas suffer severe TB related morbidity and mortality, and a large proportion of cases are diagnosed solely on the basis of medical history and clinical examination [4,6]. In Ethiopia, childhood TB is still a major cause of hospital admission and death [5,6]. Surveillance systems to monitor TB outcome in children are very important in order to know the risk factors associated with an unfavorable outcome [6]. The most critical need is for improved capability to confirm diagnosis in the developing world. Several authors have recommended a prompt and efficient identification of source of transmission, family screening and early initiation of treatment for the prevention and control of tuberculosis in children [5,6].

Many DOTS experiences in developing countries have been reported [7-11]. However, experience in a rural hospital and information on the differences between children and adults are limited. For instance, in the present study, we sought to describe the experience of tuberculosis according to the TLCP in a rural zone in Southeast Ethiopia over a period of 10 years (1998 to 2007). Furthermore, we compare childhood TB with adult TB, and identify risk factors associated with defaulting and dying during TB treatment.

## Methods

### Setting

The GRH is a 135-bed rural general hospital located in West-Arsi zone, 250 Km south of Addis Ababa. The GRH is a private mission hospital. Due to an inadequate transportation network, the catchment area of the GRH is restricted to approximately 75,000 inhabitants. Most of the population live in a rural setting and work in agriculture and farming.

### Study design and data collection

The TLCP adopted the WHO recording and reporting guidelines recommended in 1992 [2,3]. Briefly, patients with signs and symptoms suggestive of TB are screened for confirmation of the diagnosis and initiation of treatment. Patients with symptoms compatible with pulmonary tuberculosis (PTB) submit three sputum samples. Patients with at least two positive smears are considered to have smear-positive PTB; those with three negative smears are treated with antibiotics and then re-evaluated. These latter patients are considered to have smear-negative PTB if they had a chest x-ray suggestive of PTB and did not respond to a course of antibiotics. The diagnosis of extra-pulmonary TB (EPTB) is usually made clinically according to TLCP guidelines [2,3]; biopsies of lymphadenopathies, lumbar puncture, pleural and peritoneal effusion puncture were usually performed.

Patients who are diagnosed with TB at GRH were: 1) admitted to hospital in the case of seriously ill PTB or EPTB patients, 2) registered at the GRH TB clinic or 3) referred to TB clinics near their home. During the period of study (1998 to 2007), all of the patients were included in the DOTS programme. The patients were usually admitted to hospital for two months (intensive phase). The cost of hospitalization was 3 times lower than in adults admitted for other reasons. In addition, the children's admission fee was 4 times lower than that of adults. The food was provided by the hospital. After being discharged, the patients could continue to attend the GRH TB clinic or were transferred to TB clinics near their home. During the intensive phase the patients took their medication daily under the direct observation of a health worker. Patients who lived more than an hour's walk from the GRH TB clinic were provided with free accommodation in a house near the TB clinic for two months. During the continuation phase the drugs were given monthly, and were self-administered. There were three categories of treatment regimens: (1) short course chemotherapy for smear-positive PTB and seriously ill smear-negative PTB and EPTB cases, (2) retreatment regimen, (3) short course chemotherapy for smear negative PTB and EPTB patients who were not seriously ill. However the scheme changed during the period of study as shown in Table 1. TB treatment is free for patients.

**Table 1:** Scheme of treatment according to type of tuberculosis following the Tuberculosis and Leprosy Prevention and Control Programme in Ethiopia.

	2005 Guidelines	2002 Guidelines	1999 Guidelines
**Category I**: Short course chemotherapy for smear-positive PTB and seriously ill smear-negative PTB and EPTB cases	Adults 2(ERHZ)/6(EH) Children < 7 years 2 (SRHZ)/4(RH) Children ≥ 7 years 2 (ERHZ)/4(RH)	Adults 2(ERHZ)/6(EH) or 2(SRHZ)/6(EH) Children < 7 years 2 (SRHZ)/4(RH) Children ≥ 7 years 2 (ERHZ)/6(EH)	Adults 2(SRHZ)/6(TH) or 2(ERHZ)/6(TH) Children 2(SRHZ)/6(TH)
			
**Category II**: Retreatment regimen	2SERHZ/1ERHZ/5 (ERH)3		
**Category IIIa**: Short course chemotherapy for smear negative PTB, EPTB who are not seriously ill	Adults 2 (RHZ)/6 (EH) Children < 7 years 2 (RHZ)/4 (RH) Children ≥ 7 years 2 (RHZ)/6 (EH) or 2 (RHZ)/4 (RH)	Adults 2 (RHZ)/6 (EH) Children < 7 years 2 (RHZ)/4 (RH) Children ≥ 7 years 2 (RHZ)/6 (EH)	
**Category IIIb**: Long course chemotherapy for smear negative PTB, EPTB who are not seriously ill			Adults and Children 2 (STH)/10 (TH)

PTB: pulmonary tuberculosis; EPTB: extrapulmonary tuberculosis; H, isoniazid; R, rifampicin; S: streptomycin; Z: pirazinamide; E: ethambutol; T: thioacetazon

The health workers completed the case notification forms, which include details of age, sex, weight, disease status (new case, relapse, return after default, treatment failure, transferred into the GRH), type of TB (smear-positive PTB, smear-negative PTB, EPTB), treatment regimen, treatment outcome (cured, completed treatment, defaulter, failure of treatment, died or transferred out), immunodeficiency virus (HIV) status and other clinical-epidemiological data (such as the score on the chart for diagnosis of TB in children, other relatives with TB, and previous TB diagnosis). The HIV test was only performed in patients with other clinical signs suggestive of HIV infection before 2007 because the HIV test was not generally available. During 2007, the HIV test was done in almost all cases. Institutional ethical clearance was obtained from the Research and Publication Committee of the GRH and the Health Unit and Ethical Review Committee of the Ethiopian Catholic Secretariat.

### Statistical analysis

The epidemiological and clinical data were entered in an Excel 97 program. Data was analysed using the SPSS, version 12, statistical package. Continuous variables are given as medians and interquartile range (IQR). Fisher's exact test, Student's t test and Kruskal-Wallis test were used for comparison where appropriate. Each category was analysed comparing it to the rest of the cases. Crude odds ratios (OR) and 95% confidence intervals (CI) were used for the interpretation of the univariate analysis. Significant univariate predictors (p < 0.05) of mortality and default from treatment were included in a stepwise logistic regression model to identify independent predictors (adjusted odds ratios (AOR) and 95% CI).

## Results

Over the 10-year period from 1998 to 2007, 2225 patients with all forms of TB were registered for treatment. The decrease in the number of registered TB cases from 381 in 1998 and 310 in 1999 to 156 in 2000 correlated with the segregation of part of a rural zone, because of decentralisation of the DOTS strategy in the region (Table 2). Two thousand one hundred and twenty eight patients (95.5%) were newly diagnosed and 49 (2.2%) had been treated previously for TB, with no marked differences throughout the years (Table 3). Twenty-nine percent had smear-positive PTB, 36.7% smear-negative PTB and 34.3% EPTB, and no differences were seen throughout the study period (Table 3). The type of EPTB (n = 762) was: TB lymphadenitis in 411 (53.9% of all EPTB cases), osteoarticular TB in 171 (24.4%), abdominal TB in 112 (14.7%), and TB meningitis in 26 (3.4%) (Table 3).

**Table 2:** Evolution of forms of tuberculosis (TB), case definitions, tuberculosis in children, and treatment outcome during 10-year period of study.

	1998	1999	2000	2001	2002	2003	2004	2005	2006	2007
All forms of TB, n	381	310	173	156	171	190	231	224	202	187
Smear-positive PTB, %	18.6	23.2	31.8	42.9	35.1	31.6	39.4	23.7	28.7	31.6
Smear-negative PTB, %	53.5	44.2	32.4	18.6	31.0	29.5	25.6	27.9	34.7	35.8
EPTB, n %	27.8	32.6	35.8	38.5	33.9	38. 9	35.1	38.4	36.6	32.6
Case definitions										
New cases, %	95.7	96.4	96.5	95.7	95.6	96.1	93.5	96.0	96.8	94.7
Relapse, %	0.9	2.2	2.0	3.2	2.2	1.0	2.3	3.5	2.6	3.5
Transfer in, %	2.6	0.9	1.5	0	1.7	1.8	3.2	0.6	0.6	1.8
Other, %	0.9	0.4	0	0	0.4	1.0	1.0	0	0	0
Tuberculosis in children, %	61.7	54.5	42.2	32.7	45.0	43.2	43.9	49.6	38.6	28.0
Hospital admission, %	33.9	15.5	20.2	38.5	51.5	54.7	42.4	58.9	46.5	38.5
HIV status										
Negative, %	-	-	-	-	0.6	8.9	5.6	10.3	19.3	79.7
Positive, %	0.5	0.3	0.6	3.2	2.3	1.1	3.0	0.9	2.5	4.8
Unknown*, %	99.5	99.7	99.4	96.8	97.1	90.0	91.3	88.8	78.2	15.5
Treatment outcome										
Cured, %	13.9	15.5	26.6	23.1	17.5	17.4	25.1	17.0	17.8	17.5
Treatment completed, %	46.7	48.7	59.0	50.6	45.0	52.1	50.6	46.9	52.5	53.0
Transfer out, %	15.2	17.7	-	5.1	15.2	17.4	8.2	17.9	14.9	21.9
Death, %	4.3	3.2	2.9	9.0	10.5	5.8	6.5	7.1	5.0	2.7
Failure, %	-	1.0	0.6	0.6	-	-	-	-	-	-
Defaulted, %	19.8	13.9	11.0	11.5	11.7	7.4	9.5	11.2	9.9	4.9

* not done; HIV: human immunodeficiency virus; PTB: pulmonary TB; EPTB: extrapulmonary TB

**Table 3:** Differences in epidemiological data, case definitions, forms of tuberculosis (TB), and treatment outcome between paediatric and adult patients [>14 years].

	Total (%)	Paediatric patients (n = 1029) (%)	Adult patients (n = 1194) (%)	P value
Epidemiological				
Sex, female	1145 (51.5)	553 (53.7)	592 (49.6)	0.05
Hospital admission	860 (38.7)	408 (38.7)	452 (37.9)	0.5
				
HIV status				
Negative	242 (10.9)	51 (5.0)	191 (16.0)	<0.001
Positive	38 (1.7)	4 (0.4)	34 (2.8)	<0.001
Unknown*	1943 (87.4)	974 (94.7)	969 (81.2)	<0.001
				
Case definitions				
New cases	2126 (95.5)	1003 (97.5)	1123 (94.1)	<0.001
Relapses	48 (2.2)	7 (0.7)	41 (3.4)	<0.001
Other	10 (0.4)	5 (0.5)	5 (0.4)	0.2
Transfer in	37 (1.7)	13 (1.3)	24 (2.0)	0.4
				
Forms of TB				
Smear-positive PTB	646 (29.0)	132 (12.8)	514 (43.0)	<0.001
Smear-negative PTB	815 (36.4)	556 (54.0)	259 (21.7)	<0.001
All forms of EPTB	762 (34.3)	341 (33.1)	421 (35.3)	0.3
TB lymphadenitis	411 (19.5)	189 (19.1)	222 (19.8)	0.4
TB osteomyelitis	171 (8.1)	78 (7.9)	93 (6.1)	0.5
TB abdominal	112 (5.0)	39 (3.8)	73 (7.8)	0.4
TB meningitis	26 (1.2)	22 (2.2)	4 (0.4)	<0.001
TB pleural	19 (0.9)	3 (0.3)	16 (1.4)	0.01
Other EPTB	23 (1.1)	10 (1.0)	13 (1.2)	0.6
				
Treatment outcome				
Cured	402 (18.1)	99 (9.6)	303 (25.4)	<0.001
Treatment completed	1082 (48.7)	584 (56.8)	498 (41.7)	<0.001
Defaulted	254 (11.4)	143 (13.9)	111 (9.3)	0.001
Death	118 (5.3)	40 (3.9)	78 (6.5)	0.008
Failure	5 (0.2)	0	5 (0.4)	0.2
Transfer out	300 (13.5)	126 (12.2)	174 (14.6)	0.1
Unknown	63 (2.8)	37 (3.6)	26 (2.1)	0.5

* not done; HIV: human immunodeficiency virus; PTB: pulmonary TB; EPTB: extrapulmonary TB.

The median age was 17 years (IQR: 7 - 27) and 51.4% were male. Of the 2225 patients registered, in two cases the age was not recorded and 1029 (46.3%) were children (under 15 years old). The median age of childhood TB was 6 years (IQR: 1.5 - 11), and 34.1% were under the age of 3 years. In adults the median age was 26 (IQR: 20 - 36), and 67.3% of them were under the age of 31 years. Thirty-eight patients (1.7%) were HIV positive. The percentage of patients tested for HIV was 12.3%. During the period of study the number of patients tested for HIV status increased, especially during the last year. Of all the cases, 38 patients (1.7%) were HIV positive, and amongst the patients tested, 13.6% were positive.

### Differences between childhood and adult TB

Of the 646 patients with smear-positive PTB, 132 (20.4%) were children; of the 816 with smear-negative PTB, 556 (68.2%) were children; and of the 763 with EPTB, 341 (44.8%) were children. Table 3 shows the differences in the epidemiology, case definitions, forms of TB, and treatment outcome between paediatric and adult patients. Significant differences were found as regards newly diagnosed TB cases (97.5% vs. 94.1%; p < 0.001), and relapses (0.7% vs. 3.4%; p < 0.001) between paediatric and adult patients. Both the number of patients tested for HIV and the number found to be HIV positive were less in paediatric cases than in adults (0.4% and 5.4% vs. 2.8% and 18.8%, respectively; p < 0.001). In childhood TB, smear-positive PTB was significantly less frequent, whereas smear-negative PTB and TB meningitis were more common. The number who defaulted from treatment was higher in paediatric (13.9%) than in adult patients (9.3%) (p = 0.001). The mortality rate was lower in paediatric patients (3.9%) than in adults (6.5%) (p = 0.008).

### Treatment outcome

The treatment outcome reports were not available (unknown) in 2.8% (15.8% in 1998, and 2.1% in 1999). The transfer out rate was 13.5%, with no marked differences throughout the years. The failure rate was 0.2%. The average treatment success rate (cured plus treatment completed) was 66.9%, with no marked differences throughout the years. In smear-positive PTB cases, the treatment success rate was 71.9% (cured: 60.9%, and treatment completed: 11.0%).

### Defaulting from treatment

The defaulting from treatment rate was 11.4%, and decreased from 16.8% in 1998 to 4.9% in 2007. Of the 300 patients who defaulted from treatment, 37.1% did so by the end of the intensive phase. The median number of days before defaulting was 90 (IQR: 60-120). Results of the univariante analysis are shown in Table 4. On multivariate analysis, a higher defaulting from treatment rate was independently associated with TB meningitis (AOR: 2.8; 95% CI: 1.2-6.6), and smear-positive PTB was less frequently associated with defaulting (AOR: 0.47; 95% CI: 0.34-0.67).

**Table 4:** Univariante analysis of risk factors for treatment default and death among diagnosed tuberculosis (TB) patients.

	Treatment default			Death		
	Default* (n = 254)	**OR (95% CI)**	**p-value**	Died* (n = 118)	**OR (95% CI)**	**p-value**

Sex						
Female	116 (11.0)	1		52 (5.3)	1	
Male	138 (12.5)	1.15 (0.88-1.49)	0.3	62 (5.6)	1.05 (0.72 - 153)	0.8
Age, year						
0-14	143 (14.4)	1		40 (4.0)	1	
> 14	111 (9.5)	0.62 (0.48-0.81)	<0.001	78 (6.7)	1.70 (1.15-2.51)	0.008
Case definitions						
New cases	243 (11.8)	1.03 (0.5-1.94)	0.9	111 (5.4)	0.72 (0.32 - 1.59)	0.5
Relapses	3 (6.3)	0.49 (0.16-1.51)	0.3	3 (6.3)	1.15 (0.35 - 3.78)	0.6
Forms of TB						
Smear-positive PTB	45 (7.0)	0.47 (0.34-0.67)	<0.001	40 (6.3)	1.24 (0.83 - 1.83)	0.3
Smear-negative PTB	113 (14.5)	2.33 (1.79-3.03)	<0.001	47 (6.0)	1.19 (0.81 - 1.74)	0.4
EPTB	96 (12.9)	1.18 (0.90-1.54)	0.2	31 (4.2)	0.66 (0.47 - 1.01)	0.07
TB lymphadenitis	48 (12.1)	1.04 (0.74-1.45)	0.9	6 (1.5)	0.22 (0.09 - 0.52)	<0.001
TB osteomyelitis	18 (10.7)	0.88 (0.53-1.45)	0.7	7 (4.2)	0.73 (0.33 - 1.61)	0.5
TB abdominal	18 (14.3)	1.27 (0.74-2.18)	0.4	9 (8.0)	1.55 (0.76 - 3.16)	0.3
TB meningitis	8 (30.8)	3.44 (1.51-7.84)	0.006	4 (15.4)	3.22 (1.09 - 9.52)	0.04
TB pleural	4 (21.1)	2.02 (0.69-5.84)	0.3	3 (15.8)	3.18 (0.95 - 11.5)	0.06
HIV status						
Negative-Unknown	246 (11.6)	1		109 (9.2)	1	
Positive	8 (21.1)	2.03 (0.94-4.14)	0.07	9 (23.7)	5.74 (2.64-12.2)	<0.001

* n (%); Percentage out of total registered for treatment

### Mortality

Of the 2163 patients diagnosed with TB whose outcomes were available, 112 (5.3%) died. The median survival time from time of diagnosis for those who died was 28.5 days (IQR: 9.75-59.5). Paediatric patients who died had significantly shorter survival than adult patients (median 10 [IQR:3-34.5] vs. 37 days [IQR:18-65], respectively, P < 0.001). Patients who died were significantly older than those who survived (median age 22 [IQR: 0-35] vs. 17 [IQR: 7-26] years, respectively, P < 0.001). Results of the univariate analysis of risk factors for death among patients diagnosed with tuberculosis are shown in Table 4. On multivariate analysis, increased mortality was associated with adults (AOR: 1.7; CI95%: 1.1-2.5), TB meningitis (AOR: 3.6; CI 95%:1.2-10.9), and HIV infection (AOR: 4.3; CI 95%:1.9-9.4); moreover, decreased mortality was associated with TB lymphadenitis (AOR: 0.24; CI 95%: 0.11-0.57).

## Discussion

TB is a disease with diverse clinical manifestations. According to the TLCP, PTB should account for 80% of all TB cases (smear-positive PTB comprises 75-80% and smear-negative PTB 20-25% of all PTB cases), and EPTB 20% of all TB cases [2]. The distribution of the patients in this study according to the classification of tuberculosis was: 65.3% PTB (smear-positive, 44.2%, and smear-negative, 55.8%) and 34.3% EPTB. These results were similar to the findings of an eight-year overview (1998-2007) of TB case notification in Ethiopia, according to the TLCP [3]. However, if we consider only adult TB, PTB accounts for 64.7% of all TB cases (smear-positive PTB, 66.5% and smear-negative, 33.5%) and EPTB 35.3% of all TB cases. The percentage of PTB was slightly lower than in the TLCP [2,3] recommendations and Yassin et al's study [7] (72%) and closer to that found by Shargie EB and Lindtjørn B [8] (67.7%).

HIV infection increases the susceptibility to TB [9]. In this study the prevalence of TB and HIV co-infection was 1.7%. However, less than 10% of TB cases were tested for HIV. Only the data from the last year of study can truly reflect the HIV status. In the last year of the study period, the prevalence reached 5%. Studies in sub-Saharan Africa have shown that the prevalence of TB and HIV co-infection is more than 5% [10,11], and according to the WHO report the prevalence of co-infection was 11% [1]. In our study, the patients came from rural areas and the prevalence was lower.

The reported percentage of all TB cases occurring in children varies from 3% to more than 25% depending on the country [12,13]. This study shows that nearly 43% of registered TB cases in GRH were children aged 14 years or under. The largest reported number of cases of childhood TB notified in sub-Saharan Africa is 39%, from a poor and crowded urban population in South Africa [14]. This survey probably represents an over-estimate of the real burden of TB in Ethiopian children [3,13]. In a Malawi study [14], a mission hospital, like the GRH, in a rural district recorded significantly higher rates of TB than did the district hospital. Mission hospitals, some of which have paediatric services, and probably more adequate clinical and diagnostic services, are likely to detect a higher rate of childhood TB, as did the GRH. The high proportion of childhood TB compared to adult TB may be explained by the higher rate of admission of children to GRH (60%) since it is a missionary hospital. Many adults are diagnosed outside the hospital, whereas children would go to this hospital to be diagnosed because it was cheap compared to other clinics and it was possible to do a chest-X-ray. Moreover, the high proportion of childhood TB should be a good incentive for our hospital to review diagnostic procedures in children. It is often very difficult to diagnose TB in children and it may be over-diagnosed even though we followed national guidelines.

The predominant types of childhood TB were smear-negative PTB followed by EPTB, with smear-positive PTB being the least common. This would be expected because of the acknowledged difficulties involved in confirming PTB in children and young adults in samples of expectorated sputum [3,13-16]. Smear-negative PTB cases accounted for 54% of all childhood TB cases, EPTB for 33.1% and smear-positive PTB 12.8%. The incidence of TB in HIV-infected African children is low compared to HIV-infected African adults [17]. In this study the HIV prevalence was low among children with TB compared to adults with TB.

In the patients described here, the treatment success rate was about 70%. This is lower than previously report by the TLCP in Ethiopia (from 78% to 85%) [3]. More than 40% of cases were admitted to hospital. The admitted cases are more seriously ill patients, and after the initial phase of treatment are discharged and transferred out to their TB clinic. In these patients it is impossible to know if they are cured or if they complete treatment.

In this study, the default rate (11.3%) falls somewhere between the rate reported in the study by Yassin et al [4] in Ethiopia (6%), and that found by Shargie EB and Lindtjørn B [18] (20%). However, there was a significant improvement over the 10-year study period since the rate fell from 19.8% in 1998 to 4.9% in 2007. The defaulter rate declined in other studies in Ethiopia, for example from 24% in 1996 to 6% in 2004 in Yassin et al's [7] study or from 38% to 18% in Shargie EB and Lindtjørn B's [8] study. The likely causes of this drop are the short treatment regimens used during the last years of the study and the decrease in distance between the homes and treatment centre throughout the period of study. Patients with smear-positive PTB were more likely to complete treatment, possibly because their illness is more severe and symptomatic [19-22]. The higher defaulting rate among the TB meningitis patients is probably due to their more severe illness and neurological deficits. The high prevalence of default could be partially due to cases of death at home, especially in cases that occur after the intensive phase of treatment.

This study shows that the death rate (5.3%) was lower than in other studies conducted in sub-Saharan African countries (about 23%) [23]. The reason for this is not clear; defaulter rates in this study were higher, and it is possible that a large number of deaths are hidden in this outcome category. TB in adults, TB meningitis and HIV infection were associated with increased risk of death.

These findings are subject to at least five limitations. First, sociological data were not available, and the information that was available, recovered from registers, may be imprecise in some cases. Second, we were unable to collect information about HIV infection, which might have affected the likelihood of a poor outcome. Testing for HIV was performed only occasionally before 1998 and usually only positive results were annotated, with no negative requests being recorded. During the last year, the test was performed in nearly all the cases following TLCP recommendations to apply the procedure to the majority of cases of TB. Third, the GRH is a private hospital and patients came from other health facilities to be diagnosed. Fourth, the patients were then admitted to hospital and after finishing the intensive phase transferred out to their health facilities. Thus failure and death were not detected. Fifth, the treatment regimens changed during the period of study and this may contribute to the results found.

## Conclusions

Despite these limitations, several conclusions may be drawn from our results. First, the registration of TB cases can be useful to understand the epidemiology of TB in local health facilities. Second, the percentage of treatment defaulters was higher in paediatric than in adult patients and the mortality rate was lower in paediatric patients than in adults. The diagnosis of childhood TB in hospitals with poor resources needs to be vastly improved. Finally, the defaulting from treatment rate has declined, and the determinants that contribute to defaulting should be included in control programmes.

## Competing interests

The authors declare that they have no competing interests.

## Authors' contributions

JMR was the primary researcher, conceived the study, designed, participated in data collection, conducted data analysis and drafted the manuscript for publication. FR conceived the study and participated in its design and coordination. AT assisted in data collection and preparation of the first draft of the manuscript. All authors read and approved the final manuscript.

## Pre-publication history

The pre-publication history for this paper can be accessed here:

http://www.biomedcentral.com/1471-2458/10/215/prepub
